# 

**Published:** 1888-12-15

**Authors:** 


PERMANENTLY ENLARGED TO 32 PAGES.

PRICE ONE PENNY.

No. 116, Vol. V.—December 15th, 1888.

[Registered at the General Post Office as a Newspaper.]

Per Annum, 6s. 6d., post free.

Monthly Parts, 6d.; post free, 7½d.

Ditto per Ann. 7s. 6d., post free.

### THE HOSPITAL

A Weekly Institutional Journal of

